# GASTRIC SLEEVE SURGERY AS A NEW CLINICAL INDICATION FOR SURGICAL GASTROSTOMY AFTER FAILURE OF ENDOSCOPIC APPROACH IN PATIENTS WHO NEED LONG-TERM ENTERAL NUTRITION

**DOI:** 10.1590/0102-6720201700030015

**Published:** 2017

**Authors:** Gonçalo NUNES, Rita BAROSA, Carla Adriana SANTOS, Jorge FONSECA

**Affiliations:** 1 Hospital Garcia de Orta, Gastroenterology Department, GENE - Artificial Feeding Team, Almada, Portugal

**Keywords:** Obesity, Bariatric surgery, Enteral Nutrition

## INTRODUCTION

Percutaneous endoscopic gastrostomy is currently the gold standard method for long-term enteral feeding in patients with persistent dysphagia owing to oncologic and neurologic disorders^1^. However, despite its safety and practical execution, some limitations persist, especially when obstructive lesions prevent endoscopic gastric access or abdominal wall transillumitation is hampered by obesity, ascites, previous abdominal surgery or visceral interposition^1^.

## CASE REPORT

A 60 years old man previously submitted to gastric sleeve bariatric surgery was admitted to the neurosurgery department of our institution after acute trauma that resulted in fracture of the 7^th^ cervical vertebra and spinal cord injury with tetraparesia. The patient was operated with fixation of bone lesions but was further admitted to intensive care unit for mechanical ventilatory support. Tracheostomy was performed and a nasogastric tube was passed in order to start enteral feeding. Due to persistent dysphagia he was referred to our artificial feeding team for percutaneous endoscopic gastrostomy. Upper GI endoscopy showed a small tubular stomach, difficult to distend and examine. Abdominal wall transillumitation (diaphanoscopy) was not obtained, preventing a safely percutaneous cannulation of the stomach. The patient underwent surgical laparoscopic gastrostomy a few days later without early complications. At the present, he maintains follow-up in our artificial feeding outpatient clinic with no long-term complications of gastrostomy feeding.

## DISCUSSION

Bariatric surgery is more and more used as a major tool on the treatment of severe obesity. Globally, surgical procedures may be classified in one of three groups: restrictive procedures (as the adjustable gastric band or the laparoscopic sleeve gastrectomy), malabsortive procedures (as gastric bypass) or procedures combining both gastric restriction and malabsorption (as the biliopancreatic diversion with duodenal switch)^2^. Although severely obese patients are not considered as a typical candidate to long-term tube feeding, they may develop any of the dysphagia causes that usually lead to gastrostomy. More, these patients frequently present severe vascular disease and are prone to develop stroke episodes, even after bariatric surgery. Growing need for gastrostomy after bariatric surgery is to be expected in the years to come.

Naturally, percutaneous endoscopic gastrostomy is impossible after procedures like gastric bypass, where the stomach is not accessible to endoscopic examination. Sleeve gastrectomy is a restrictive procedure that includes removal of most of the stomach, especially fundus that contain the appetite stimulant ghrelin secreting cells, leaving only a thin gastric tube between esophagus and duodenum that contributes to reduce food ingestion^3^. Gastric endoscopy is possible after gastric sleeve surgery, but it reduces above 80% of stomach volume^4^ affecting lumen distension and making abdominal wall transillumitation an extremely difficult task. To the best of our judgment, it is not advisable to try the endoscopic approach for creating a gastrostomy after this restrictive procedure. In patients who need long-term enteral nutrition, gastric sleeve surgery emerges as a new clinical indication for SG, not previously reported in literature.
